# The Effect of Zn and Zn–WO_3_ Composites Nano-Coatings Deposition on Hardness and Corrosion Resistance in Steel Substrate

**DOI:** 10.3390/ma14092253

**Published:** 2021-04-27

**Authors:** Channagiri Mohankumar Praveen Kumar, Manjunath Patel Gowdru Chandrashekarappa, Raviraj Mahabaleshwar Kulkarni, Danil Yurievich Pimenov, Khaled Giasin

**Affiliations:** 1 Department of Chemistry, PES Institute of Technology and Management, Shimoga-577204, Visvesvaraya Technological University, Belagavi 590018, India; praveen.cm@pestrust.edu.in; 2 Department of Mechanical Engineering, PES Institute of Technology and Management, Shivamogga, Visvesvaraya Technological University, Belagavi 590018, India; 3 Centre for Nanoscience and Nanotechnology, Department of Chemistry, KLS Gogte Institute of Technology, Belagavi 590006, India; ravirajmk@git.edu; 4 Department of Automated Mechanical Engineering, South Ural State University, Lenin Prosp. 76, 454080 Chelyabinsk, Russia; danil_u@rambler.ru; 5 School of Mechanical and Design Engineering, University of Portsmouth, Portsmouth PO1 3DJ, UK; Khaled.giasin@port.ac.uk

**Keywords:** electrodeposition, Zn–WO_3_ composite, XRD, corrosion behavior

## Abstract

Pure Zn (Zinc) and its Zn–WO_3_ (Zinc–Tungsten trioxide) composite coatings were deposited on mild steel specimens by applying the electrodeposition technique. Zn–WO_3_ composites were prepared for the concentration of 0.5 and 1.0 g/L of particles. The influence of WO_3_ particles on Zn deposition, the surface morphology of composite, and texture co-efficient were analyzed using a variety of techniques, such as X-ray diffraction (XRD) and scanning electron microscopy (SEM) with Energy Dispersive X-ray analysis (EDX). Higher corrosion resistance and microhardness were observed on the Zn–WO_3_ composite (concentration of 1.0 g/L). The higher corrosion resistance and microhardness of 1.0 g/L Zn–WO_3_ nanocomposite coatings effectively protect the steel used for the manufacture of products, parts, or systems from chemical or electrochemical deterioration in industrial and marine ambient environments.

## 1. Introduction

Steels are extensively used in industries (chemical, manufacturing and marine) for versatile engineering applications, owing to their low cost and potential material properties [1,2,3]. Excellent mechanical strength and hardness, machinability, weldability and formability have made steel alloys an ideal candidate for structural applications [1]. Steels used in functional parts of many industrial applications (power plants, automotive, construction) eventually undergo corrosion due to their low thermodynamic stability [1,4,5,6,7,8,9]. The materials undergo corrosion, which inhibits the proper functioning of parts, which fails parts during their service life [10,11,12]. In the United States, it was estimated that the cost of corrosion destruction is 6.2% of the gross domestic product (GDP) and accounts for more than 3% of the world GDP [13,14]. Therefore, studies on the development of corrosion-resistant materials and processing methods are urgently needed with industrial and economical relevance.

Although a few metallic materials, such as gold, silver, and platinum, possess excellent corrosion resistance characteristics, their use is restricted for engineering-based structural applications owing to their high cost and poor mechanical strength [15]. In recent years, two popular processing routes—the addition of alloying elements and coatings—are widely practiced industry methods to develop corrosion-resistant materials [16,17]. Numerous research efforts were made to protect metal surfaces (creating protective passive films) against corrosion with the well-known addition of the alloying elements (traces of chromium, nickel, molybdenum and so on) method [16,18,19,20,21]. In the alloying process, the presence or formation of precipitates might result in localized corrosion, which affects the overall corrosion resistance [22,23,24]. In addition, the alloy processing route as the corrosion inhabitant method is not ideally suitable for highly sensitive engineering service environments when there is a lack of high-temperature stability in base or conventional material [13,25]. In recent years, surface coating methods (electro-spark deposition, laser cladding, magnetron sputtering, thermal spray process and chemical-vapor deposition) have become practiced widely in industries [26,27]. Thermal spraying and electroplating techniques are widely applied coating techniques to protect or repair the metallic surfaces of mechanical parts [28,29]. The combination of Mo–Ni–Cr coatings is applied on the substrate surfaces of super duplex stainless steel by applying plasma spray coating techniques [30]. Thermal spray coatings are not ideally suited for complex-shaped geometries due to poor adhesion of the coating on the substrate, brittleness, undesirable mechanical strength, stresses and heat-affected zones [31,32,33]. The laser cladding process has greater potential to limit the shortcomings of thermal spray coating techniques, wherein the concentrated heat source released from the lasers to apply coating on the substrate ensures better metallurgical bonding [31,34]. A laser cladding technique was applied to coat nickel-based composite on H13 steel, which offers enhanced wear resistance and thermal fatigue properties [35]. Although laser clad deposits produced superior properties, the small spot or concentration size of lasers hinders large widths to be deposited in a single pass [36]. Magnetron sputtered molybdenum di-sulfide titanium nitride coatings on high-speed steels produced superior surface hardness [37]. The main disadvantages of magnetron sputtering are a low deposition rate and a strong decrease in the ion substrate current with an increased combination of the substrate to target distance [38,39]. Electrodeposition or electroplating techniques have recently been employed to enhance the surface properties, which inhibit corrosion and wear with the improved mechanical strength of coatings [40]. Compared to sputtering, evaporation and chemical vapor deposition methods, and electroplating or electrodeposition methods are more reliable and economical [41,42]. Composite electroplating is widely practiced in industries to limit the coating failures of the electrodeposition method in adverse environmental conditions subjected to the high potential of hydrogen (pH) and low temperature [43]. Composite electroplating techniques are best suited for the inclusion of metallic or non-metallic particles in the plated layer that enhance tribological, thermal and mechanical properties [44,45]. Therefore, experimental studies on composite-based electrodeposition or electroplating techniques could enhance the surface properties of coating materials that are of industrial relevance.

The selection of appropriate coating materials from a wide range of potential materials libraries (metals, ceramics and polymers) as a protective layer on metal surfaces to enhance the wear and corrosion properties is a tedious task for manufacturers [46]. All materials, such as Al, Ti, Ni, Cr, Mo, ZrO_2_, Al_2_O_3_, PTFE and so on, are ideal candidate materials for wear and corrosion resistance, but each material possesses different mechanical, thermal, and chemical behavior, and associated melting points [47]. The applications of zinc coatings improve wettability, which results in enhanced interfacial bonding between the materials [48]. Zinc-based coatings replace the high toxicity of cadmium coatings at low cost with better corrosion resistance, weldability and ductility [49]. The use of hard materials, namely carbides (SiC and WC) and oxides (ZrO_2_, Al_2_O_3_, TiO_2_ and SiO_2_), as second phase materials in composite coatings offers superior hardness, high-temperature oxidation resistance, and self-lubricating properties for industrial parts [42,50]. Therefore, the study of multi-layer zinc-based composite coatings (hard materials as second phase material) with electrodeposition processing routes ensures that low cost enabled better coating properties are of industrial relevance. 

In recent years, a few attempts have been made to develop the zinc and zinc-based coatings that serve as protective films on the metal substrate from corrosion. The corrosion behavior of carbon steels coated with different phosphate layers (zinc, zinc–iron and manganese-based phosphate solutions) was studied for carabiners manufacturing applications [51]. Zinc-based coated samples offered better corrosion resistance properties than manganese phosphate solutions. Zinc and zinc–SiC composite coatings were applied with the pulse electrodeposition technique to examine their corrosion resistance properties [52]. The SiC particles dispersed uniformly in the zinc matrix by filling gaps and crack-type defects, resulting in a smooth surface that finally resulted in better corrosion resistance properties. Multi-layer zinc-based composite coatings (Zn–Ni, Zn–Ni–Fe_2_O_3_, Zn–TiO_2_, Cu–Sn–Zn–TiO_2_, Zn–TiO_2_/TiB_2_) deposited on steel substrates via the electrodeposition technique resulted in better corrosion resistance [42,48,50,53,54,55]. The application of copper and zinc antifouling coatings is more beneficial for resisting corrosion by creating artificial surfaces on shipping and marine industrial parts [56]. The experimental studies reported that zinc-based antifouling coatings offer comparatively better performance with low toxicity to aquatic organisms. Therefore, zinc-based coatings are environmentally friendly and are found to be a better alternative to copper. Electrodeposited Zn–TiO_2_ nanocomposite coatings were deposited on steel substrate experimentally with a zinc sulfate bath [53]. The galvanizing coating method is widely applied in industry practice to protect the steel substrate surface [57]. However, the use of advanced coating techniques ensures the deposition of a wide range of metallic and non-metallic materials useful for different applications [58]. The zinc electroplating technique is treated as a cost-effective technique that offers better mechanical properties with a safer working environment and safer equipment [59]. Composite coating methods (electrodeposition and galvanostatic) are compared to evaluate the morphology of Zn–SiC nanocomposite coating deposits [60]. Electrodeposited coatings resulted in smaller grain sizes in composites. Tungsten trioxide (WO_3_) is a potential candidate material for zinc composite coatings because of its abundant availability and applications in preparing gas sensors [61], thin films used as pigments in paints and ceramics [62], fire resistance fabrics [63], electrochromic windows [64], and tungstates for x-ray phosphorus screens [65]. It was confirmed from the above literature review that WO_3_ and Zn-based composites are ideal materials for preparing novel nanocomposite coatings, which are ideally best suited for a wide range of engineering applications. 

To date, nickel and chromium composite coatings are applied to protect steel parts, but they are not generally recommended due to their hazardous nature. Therefore, significant attention is required to find an alternate, environmentally friendly coating material for protecting steel parts used in practical applications. Several researchers have studied WO_3_ as a reinforcement material for composites; however, no attention has been paid yet to use nano-sized Zn–WO_3_, which might improve hardness and corrosion resistance in steel substrates. The present research aims to develop nano Zn–WO_3_ coatings suitable for engineering applications (barrels, tanks, boilers, etc.) that offer a long service life, measured in terms of corrosion resistance as the prime requirement. The concentration of tungsten trioxide (WO_3_) nanoparticles on steel substrate depositions were examined for their surface morphology and corrosion resistance property on composite films. Electrodeposition coating techniques were selected due to their simplicity, low cost, and deposition of thin, high-quality films, ensuring precise control over thickness, reducing material waste on industrial parts possessing different shapes compared to other coating methods [62]. The WO_3_ nanoparticles added to Zn composite coatings viz. an environmentally friendly bath solution (10% diluted H_2_SO_4_ + 90% diluted NaHCO_3_), which is less harmful and potentially stable enough to store and use, which is not yet reported in the literature. A systematic study of Zn and Zn–WO_3_ composite coating (i.e., WO_3_ concentration of 0.5 and 1.0 g/L) was examined with electrochemical test results (polarization curves, electrochemical impedance spectroscopy) and validated with non-electrochemical tests (XRD, SEM). The impact of WO_3_ nanoparticles on hardness and corrosion resistance behavior was discussed for their practical usefulness.

## 2. Materials and Methods

**Figure 1:**
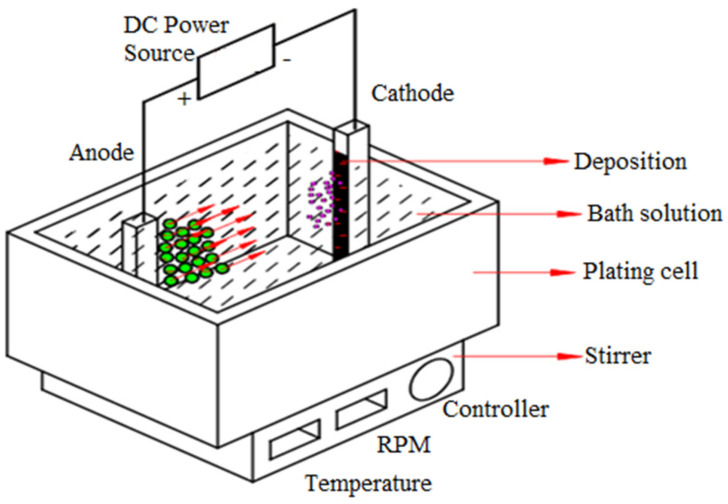
Experimental setup for composite electrodeposition.

Table 1 describes the details of the composition of the electroplating bath for zinc, zinc composites, particle concentrations, and operating parameters. Figure 1 shows the experimental setup of the electrodeposition technique. Hall cell studies were applied to optimize the concentration of surfactants (0.05 g/L) and WO_3_ (0.5 and 1 g/L) in the electroplating solution. The addition of dilute H_2_SO_4_ (10%) and dilute NaHCO_3_ solution maintained the desired pH of the bath solution. The WO_3_ nanoparticle was purchased from Sigma-Aldrich (Bengaluru, India). Prior to the plating experiments, the electrolytes were subjected to magnetic stirring for 24 h and the ultrasonication process ensured minimization of the agglomeration of nanoparticles. Mild steel was used as a cathode substrate material (40 mm × 4 mm × 1 mm). For deposition, zinc (99.99% purity) served as an anode material. Using DC currents, coating depositions were made viz. potentiostat/galvanostat (Model PS-618, Chemilink systems, Mumbai, India). The electrolyte was stirred continuously at 300 rpm during the deposition of the composites. After ensuring the required coating depositions, loosely adhered particles on the coated samples were washed for 5 min with distilled water. Prior to the plating experiments, the substrate material (mild steel plates or cathode) was degreased with trichloroethylene, followed by polishing with emery paper possessing different grit sizes (300–5000 mesh) and finally washed with water and air-dried. Zinc or anode surfaces activate only after dipping in 10% HCl solutions for a few seconds, followed by a water wash.

The coating surface morphology analysis was examined, subjected to JEOL-JEM-1200-EX II SEM (Tokyo, Japan). SEM coupled EDX (Tokyo, Japan) helped to determine the presence of particle content in the coated deposit. The electrodeposits (Zn, Zn–WO_3_ composites) were subjected to XRD analysis to estimate the average crystal size of coating via Philips PW 3710 XRD (Philips, Eindhoven, The Netherland) with copper Kα radiation (set at 30 mA and 40 kV). The CHI660C electrochemical workstation (measurements: potentio-dynamic polarization at aerated conditions and electrochemical impedance spectroscopy EIS) helps to examine corrosion behavior in 3.5% sodium chloride solution. To conduct deposition studies of the coatings (Zn and Zn composite) different measurements are carried out on traditional three-electrode cell, i.e., test samples (electrode as mild steel) entrenched in a Teflon holder were exposed to the corrosive medium surface area of 1 cm^2^, and platinum wire and an SCE electrode act as a counter and reference electrode. Test samples are dipped completely in a corrosive medium (3.5% NaCl and pH neutral) to establish the steady-state potential before measurements. In an open circuit, potentials are varied by ±200 mV at a scan rate of 0.01 V s^−1^ in 3.5% NaCl could help to draw Tafel polarization plots. EIS data were collected within the wide frequency ranges of 10 mHz to 100 kHz. Nyquist plots were curve fitted viz. ZSimpWin 3.21 software (Echem Software, Ann Arbor, MI, USA) based on the collected impedance data. 

## 3. Results and Discussion

This section discusses the SEM morphology and XRD patterns of WO_3_ particles analysis of WO_3_ nanoparticles’ impact on electrodeposited Zn, cathodic polarization, and cyclic voltammetric studies, characterization of Zn and Zn–WO_3_ composite deposits, and corrosion behavior of composites.

### 3.1. Particle Characterization

**Figure 2:**
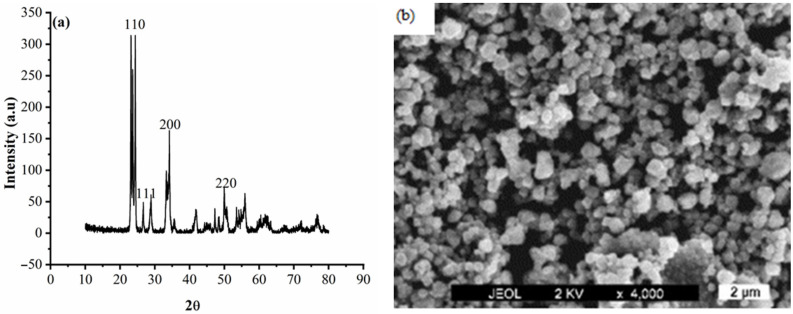
WO_3_ particles of (**a**) XRD pattern and (**b**) SEM.

The SEM morphology and XRD patterns of WO_3_ particles (purchased from the SD Fine-CHEM Limited, Chennai, India) are shown in Figure 2. In Figure 2, WO_3_ particles are seen to be agglomerated and sintered to form large crystallites. The WO_3_ particles of average crystallite size were computed from Equation (1) and were found to be 87 nm. The appearance of the crystallite surfaces can be described as smooth and uniformly distributed on the surface. The SEM images show the presence of agglomerated WO_3_ particles. The presence of agglomerated WO_3_ particles ensures better electron conductivity, and crystallographic interconnectivity [66]. 

### 3.2. Electrodeposition

**Figure 3:**
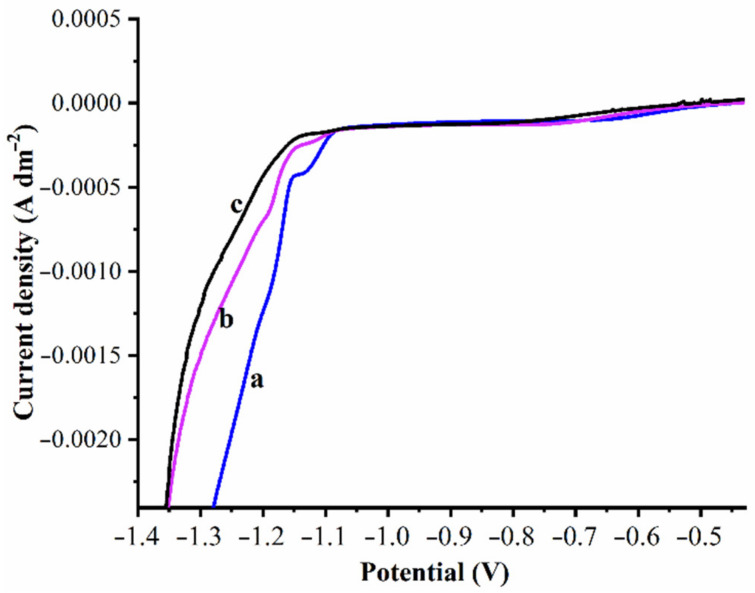
Cathodic polarization curves: (**a**) Zn (**b**) Zn–WO_3_ (0.5 g/L) and (**c**) Zn–WO_3_ (1.0 g/L) deposition.

To examine the influence of WO_3_ nanoparticles on electrodeposited Zn, cathodic polarization and cyclic voltammetric studies were carried out. Experiments were performed with increasing WO_3_ nanoparticles gradually, as 0.5 g/L, 1 g/L, 2 g/L, 3 g/L, and 5 g/L. However, after 1 g/L of the WO_3_ nanoparticle, the corrosion resistance tends to decrease. This occurs due to the increased nanoparticle concentration in the bath solution, which initiates agglomeration and hinders dispersing uniformly into the matrix or desorbing from the matrix. Cathodic polarization was carried out for bath solutions I, II, III containing 0, 0.5 and 1.0 g/L of WO_3_ nanoparticles. Measurements were taken subjected to a potential range of 0 to −1.6V, which are expressed as polarization curves shown in Figure 3. In Figure 3, curve (a) signifies the cathodic polarization of the Zn bath solution, whereas curves (b) and (c) represent the polarization curves of the Zn composite containing the solution bath of 0.5 and 1.0 g/L of WO_3_ nanoparticles, respectively. The presence of the WO_3_ nanoparticles clearly shows the curves shifted toward a higher negative potential (refer to Figure 3). The curves shifted to a negative potential is desirable for the cathode protection system [67]. In the present case, WO_3_ is an inorganic material adsorbed on the cathode surface, and this raises the reduction potential of Zn^+2^ ions. The shift of potential noticed in the case of the bath solution composed of 1.0 g/L of WO_3_ was appreciable compared to the presence of 0.5 g/L WO_3_ and the bare Zn deposition. This further suggests and supports that the adsorption of WO_3_ on the cathodic surface increases with the WO_3_ particle concentration in the bath solution during Zn deposition. This occurs due to increased nanosized WO_3_ particles, which improve the nucleation rate, which hinders the crystal growth that results in the reduced grain size of the composite. The research findings are in good agreement with the published literature [68].

**Figure 4:**
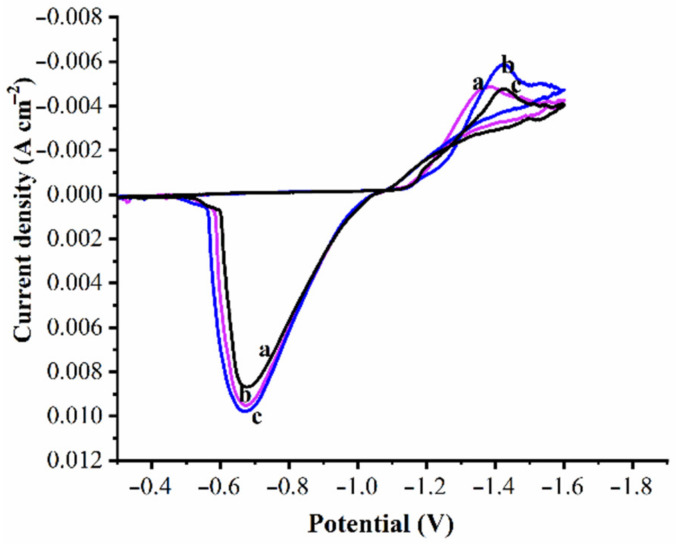
Cyclic voltammograms for (**a**) Zn (**b**) Zn–WO_3_ (0.5 g/L) and (**c**) Zn–WO_3_ (1.0 g/L) plating baths.

The cyclic voltammograms that were collected correspond to Zn and its composite bath solutions. Figure 4 shows the cyclic voltammogram curves associated with Zn and Zn–WO_3_ composite plating baths. The data that correspond to voltammograms are collected subjected to the potential range between −0.3 to −1.9 V. In Figure 4, curve (a) represents the voltammogram of the Zn bath solution, while curves (b) and (c) represent the voltammograms of Zn composite solutions II and III in 0.5 and 1.0 g/L of nanosized WO_3_ particles, respectively. The anodic portion of the cyclic voltammograms exhibited well-shaped curves for all three solutions. The anodic peak current was in the order (a) > (b) > (c). This once again indicated that, the influence of WO_3_ on the dissolution of Zn coating. This confirms that Zn composite coatings possess higher corrosion resistance properties than pure Zn coating. The results are in line with the earlier literature [69]. In the cathodic region, the curve (c) transformed toward more negative potential than the curve (a) and (b), which finally suggests a more reduced grain size of the Zn composite III coating containing 1 g/L particles. This indicates that WO_3_ particles are successfully reinforced in Zn deposition. 

### 3.3. Characterization of the Deposits

**Figure 5:**
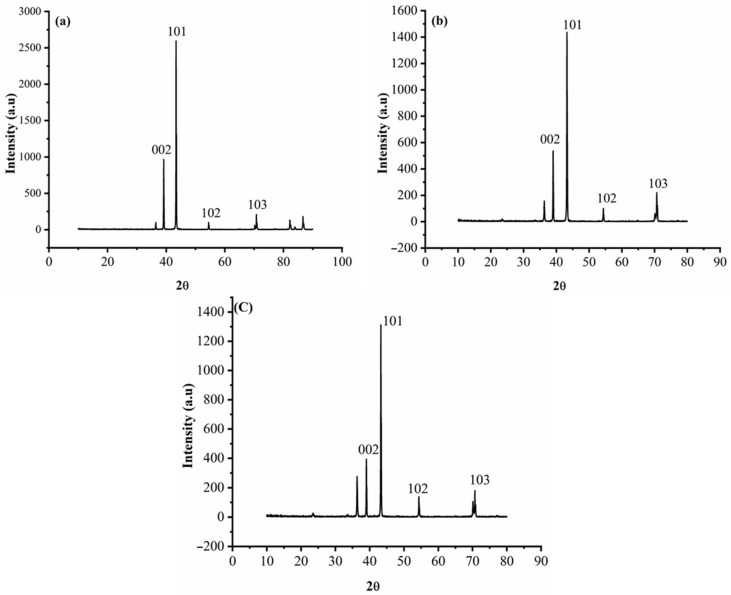
XRD pattern of (**a**) Zn (**b**) Zn–WO_3_ (0.5 g/L) and (**c**) Zn–WO_3_ (1.0 g/L) composite coating.

Figure 5 shows the XRD patterns of the Zn and Zn-composite deposits, whereas Figure 5a–c represents the XRD pattern of the bare Zn deposit, II (Zn–WO_3_ of 0.5 g/L) and III (Zn–WO_3_ of 1 g/L). The XRD graph confirms that the intensity of the diffraction lines of Figure 5c is decreased with a larger width when compared to other Zn composites (0.5 g/L) and pure Zn coating. Williamson-Hall and Sherrer’s method can be applied to determine the crystallite size [4,70]. The present work used the Sherrer’s method to determine the average crystal size of deposits as plated Zn and Zn–WO_3_ composites (Equation (1)). The average crystal size of Zn is 70 nm, and the Zn–WO_3_ composites obtained from bath solutions II and III are 59 and 55 nm, respectively. This infers that the inclusion of WO_3_ brings down the crystal size of the coating. The WO_3_ particles help as a protrusion in the metal electrolyte interface in the first stage of the electrodeposition process. This results in increased current density, which improves the nucleation rate and reduces the grain size by hindering the crystal growth of Zn in the deposits. Results of a similar nature were reported in the published literature [68].

(1) $$L_{hkl}=\frac{k\lambda }{\beta cos\theta }$$

L_hkl_ represents the average crystal size, K is the Scherrer constant kept fixed to 0.9, λ be the x-ray tube wavelength, β depicts the width of the peak in the middle of its height, and θ represents the Bragg angle.

**Figure 6:**
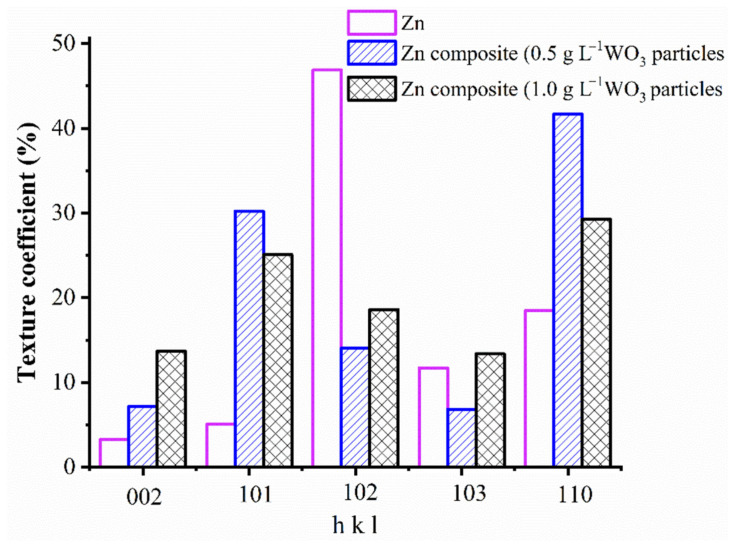
Texture coefficient of Zn and Zn–WO_3_ composite coating.

Figure 6 depicts the texture co-efficient of the Zn and Zn–WO_3_ composite coating. The significance of the texture coefficient is to ensure the preferred orientation of the crystalline plane in terms of angle 2θ where the Zn crystals are deposited. Texture coefficient calculations of electrodeposits are made from Equation (2). XRD data patterns representing the intensity of the diffraction lines were used while calculating the texture coefficients.

(2) $$T_{C}(hkl)=\frac{I(hkl)}{\sum I(hkl)}Χ\frac{\sum I_{o}(hkl)}{I_{o}(hkl)}$$

*T_C_* (*hkl*), is the texture coefficient of reflection peak. *I*(*hkl*) represents the peak intensity correspond to Zn electrodeposits and Σ*I*(*hkl*) depicts the sum of all intensities of the diffraction peaks. Index 0 implies the intensities of standard peaks of Zn powder (extracted from Joint Committee on Powder Diffraction Standards (JCPDS) file card number 87-0713). Maximum T_c_ values imply the preferred orientation of coating deposits.

Figure 6 reveals that pure zinc coating exhibited maximum orientation in <102> plane possessing minimum surface energy. However, embedded WO_3_ nanoparticles changed the preferred orientation of the zinc matrix to <110> plane for both the Zn composites. This clearly explains that forming <110> structure of the Zn layer acts as an energy barrier and, therefore, <110> preferred the orientation of both Zn composite grown on <110> plane, which is comparatively simpler compared to the rest crystal planes [71]. Note that all orientations possess a maximum texture coefficient (T_C_) value which describes the maximum number of crystallographic orientations of the Zn deposit. The WO_3_ particles in the bath solution and the matrix could impact largely on the deposit morphology and orientation. Similar findings are seen in Co–TiO_2_ composite coatings [72].

**Figure 7:**
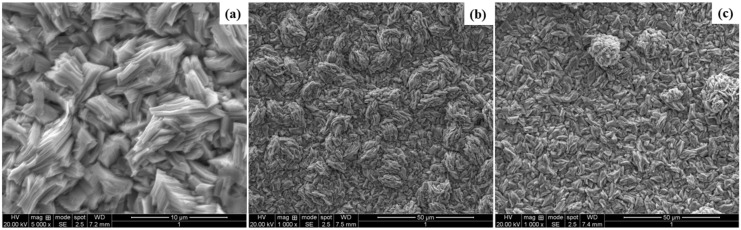
Surface micrographs of (**a**) Zn (**b**) Zn–WO_3_ (0.5 g/L) and (**c**) Zn–WO_3_ (1.0 g/L) coated samples.

The morphology of bare Zn and Zn–WO_3_ composite samples prepared from bath solutions II and III containing 0.5 and 1.0 g/L are shown in Figure 7a–c. It was observed that the pure zinc-coated sample resulted in an undesirable and improper deposition all over the substrate (refer to Figure 7b). However, morphologies of composite coated samples resulted in being uniform, compact, and smooth with appropriate crystal arrangements (refer to Figure 7c). WO_3_ nanoparticles in Zn composite coatings ensure more nucleation sites that restrict crystal growth. It was confirmed that the composite coated samples ensure a smaller grain size with uniform coating surfaces deposited on the substrate. The reduction in crystal size is attributed as per Nae-Lih Wu’s model [73]. It was confirmed from the explanation of Nae-Lih Wu’s model that the growth of crystallites is constrained owing to the interfacial boundaries between WO_3_ and Zn [74,75], which result in the reduced crystal size of WO_3_ particles. Therefore, an increased specific area is attributed to the reduced particle size. A reduced grain size is always desirable for obtaining higher microhardness and corrosion resistance in composite coatings [76,77].

**Figure 8:**
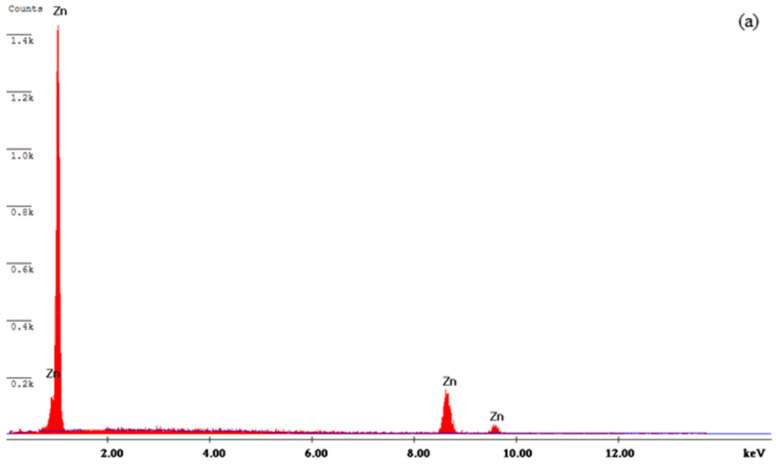

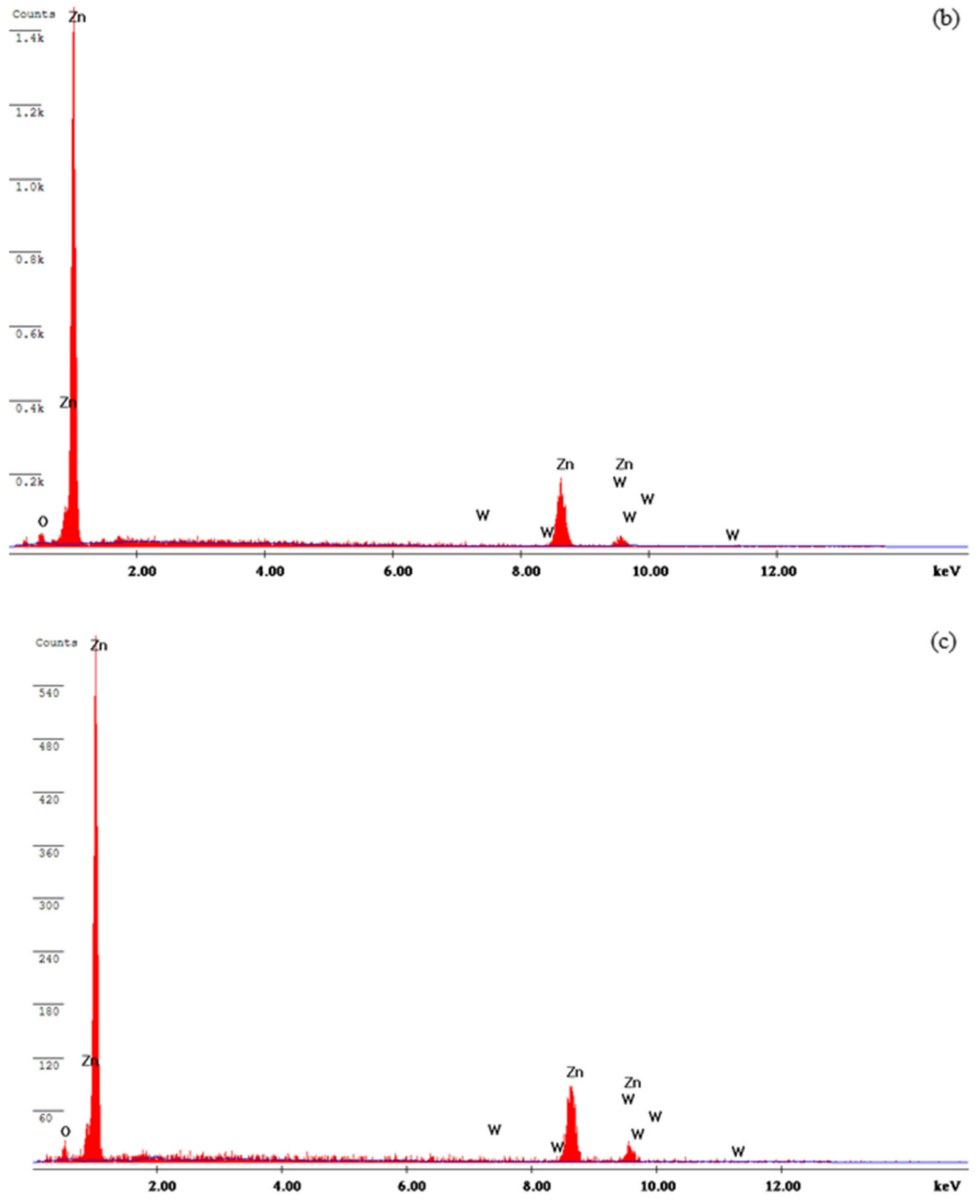
EDS spectra of (**a**) Zn, (**b**) Zn–WO_3_ (0.5 g/L) and (**c**) Zn–WO_3_ (1.0 g/L) composite coating.

The EDAX pattern of the bare Zn and Zn composite (WO_3_ nanoparticle concentrations of 0.5 g/L and 1 g/L) deposits is shown in Figure 8a–c. The composite electrodeposits show the presence of elements, i.e., W and O (refer to Figure 8b–c). This shows that the presence of WO_3_ nanoparticles can be effectively dispersed into the Zn matrix. Figure 8b,c shows the presence of WO3 nanoparticles’ inclusion in the matrix of electrodeposited coatings. Figure 8c shows increased WO_3_ particle concentration in the bath solution, which increases the number of particle incorporation at a greater level. The presence of a uniform dispersion of the WO_3_ nanoparticles is confirmed during the scanning of electrodeposited composite coatings.

**Figure 9:**
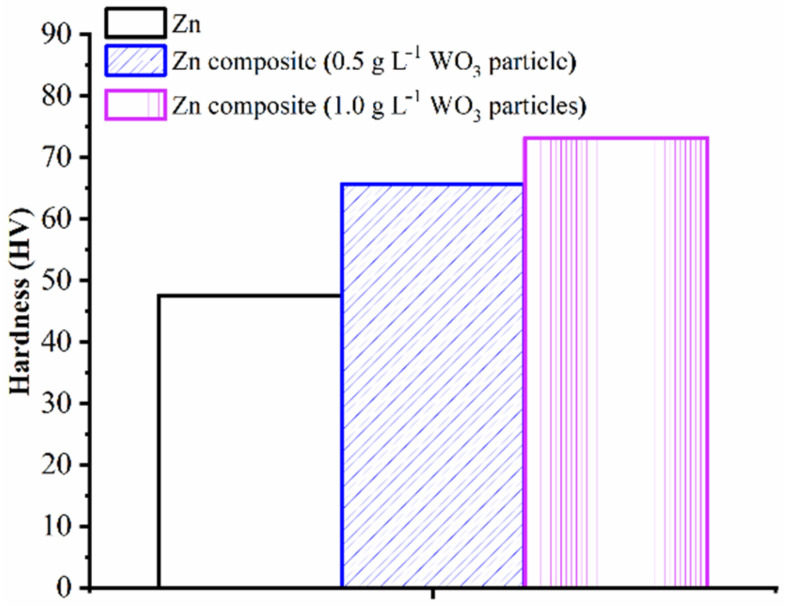
Microhardness measurements of Zn and Zn–WO_3_ deposits.

Electrodeposited coating possessing a thickness of 15 µm were subjected to microhardness examination using Vickers’s microhardness tester (Clemex digital microhardness tester, Tokyo, Japan). The microhardness tests were carried out by applying a load of 50 g for 10 s of dwell time. Figure 9 shows the average values of fifteen microhardness (five measurements on each coating sample and three replicates for each sample) measurements carried out at different locations on the deposited flat surface of each coating sample (Zn, Zn–WO_3_ of 0.5 g/L and Zn–WO_3_ of 1 g/L). Note that deviation from the average microhardness values of coatings were found to be ±0.6 HV. The Vickers microhardness of Zn and Zn–WO_3_ of 0.5 g/L and Zn–WO_3_ of 1 g/L composite electrodeposits are found equal to 47.6, 65.6 and 73.2 HV, respectively. An increase in WO_3_ particle concentration (from 0.5 g/L and 1.0 g/L) tends to increase the microhardness by 11.58% on electrodeposits (refer to Figure 9). It is important to note that WO_3_ particles of 1 g/L composite electrodeposits resulted in a 53.78% increase in microhardness compared to bare Zn coating deposits. The higher microhardness in composite coatings is attributed to finely dispersed WO_3_ nanoparticles in the matrix, which results in a fine-grained structure that restricts the easy movement of dislocation (refer to Figure 7c). Fine WO_3_ nanoparticles in electrodeposits firmly hold the Zn granules and ensure progressive nucleation growth that limits the growth of the grain size, which increases the hardness of the coatings. Similar research findings are observed in the published literature [76].

### 3.4. Corrosion Behavior of Coatings

**Figure 10:**
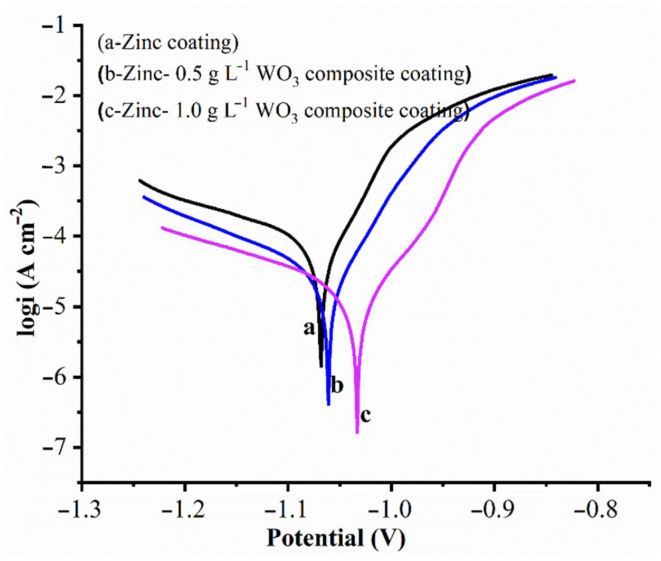
Tafel curves of Zn and Zn–WO_3_ composite coatings in 3.5% NaCl solution.

The electrochemical measurements were conducted to analyze the Zn–WO_3_ composite coatings’ quality. The Tafel extrapolation of pure Zn and its composite coatings were recorded after 5 min exposure in neutral 3.5% NaCl corrosive media and are given in Figure 10. The results that correspond to the corrosion kinetic parameters are presented in Table 2.

The composite coatings of corrosion potential values showed lesser negative values than the pure Zn coating (refer to Table 2). This justifies the potential or noble character of the composite (Zn–WO_3_) coating. The addition of WO_3_ particles in coatings influenced largely on the corrosion behavior; similar observations were reported in past literature [76]. The corrosion current that corresponds to the electrodeposited Zn coating is 6.316×10^−5^ cm^−2^ (curve a), whereas for curves (b) and (c), it is 3.118 × 10^−6^ cm^−2^ and 7.110 × 10^−7^ cm^−2^ for WO_3_ of 0.5 g/L and 1 g/L concentrations, respectively. Note that the corrosion currents (i_corr_) that correspond to Zn-composite coatings are comparatively lesser than that of the Zn coating. This could be expected for composite coatings, such that the WO_3_ nanoparticles are co-deposited on Zn. The corrosion rates of Zn, Zn–WO_3_ of 0.5 g/L and Zn–WO_3_ of 1 g/L composite electrodeposits are found equal to 32.69, 8.229, 2.803 Å min^−1^, respectively. Better corrosion resistance in composite coatings is attributed to the incorporation of WO_3_ particles which act as a physical barrier to the corrosion phenomenon by filling the gaps and micro-holes (if present) on the composite coating surfaces [68]. Note that higher corrosion resistance was obtained for the coating prepared from plating bath III. This occurs due to the increased concentration of WO_3_ nanoparticles (from 0.5 g/L and 1.0 g/L) suspended in the Zn coatings, which decrease (by 65.94%) the active surface area that undergoes the corrosion phenomenon. 

**Figure 11:**
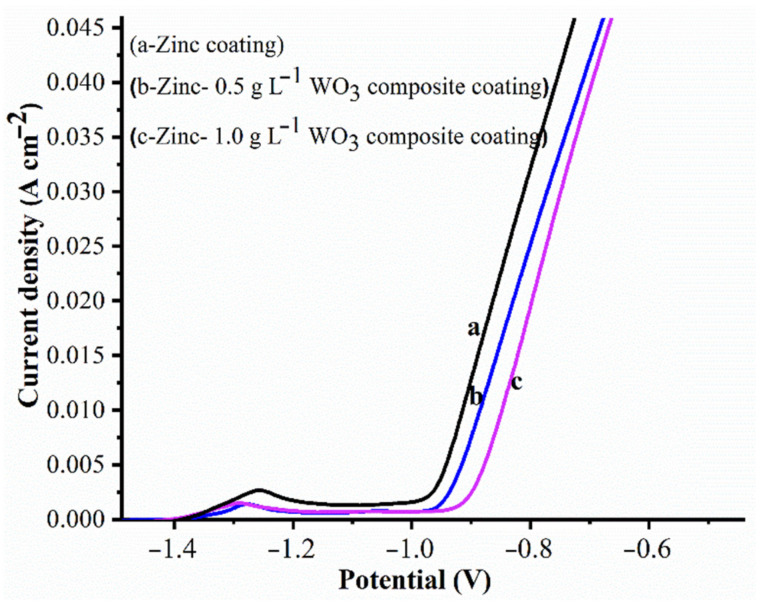
Anodic polarization curves of Zn and Zn–WO_3_ composite coatings samples in 3.5% NaCl solution.

**Figure 12:**
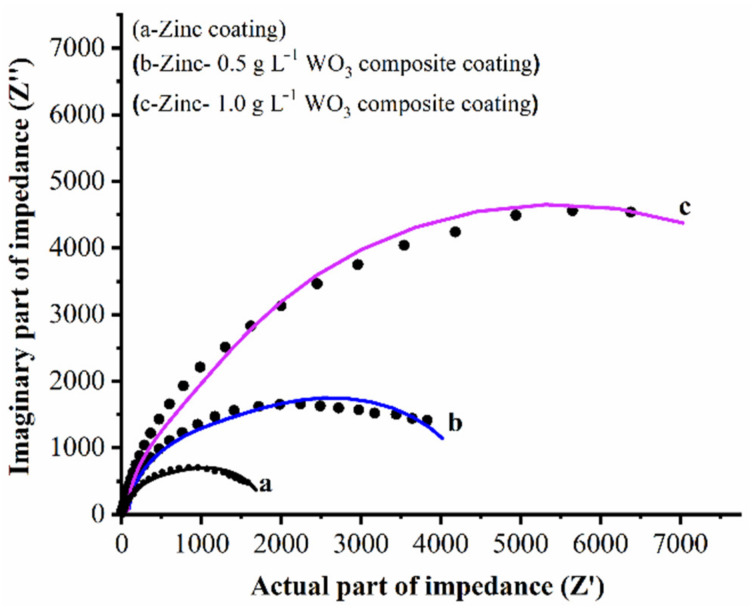
Impedance spectra of Zn and Zn–WO_3_ composite coatings in 3.5% NaCl solution.

The anodic polarization responses of Zn and Zn composite deposits fabricated from bath solutions I, II and III in 3.5% NaCl solution with varied potential ranges are presented in Figure 11. The curves are almost identical in shape, which might be due to the negligible (except at lower negative potential than the potential of Zn dissolution process) influence of WO_3_ nanoparticles in electrodepositing during the dissolution process. The reinforced WO_3_ particles held Zn deposition firmly on the steel substrate and, often, it was found to be difficult to dissolve the zinc deposition during the anodic polarization. Therefore, WO_3_ particles’ inclusion in Zn deposits offers better corrosion resistance. It was observed that the potential values are seen to be slightly less or more positive toward composite coatings (curves b and c) compared to the pure Zn coating (curve a) at all current density values. Zn oxidation occurs at a less negative potential, whereas a higher concentration of WO_3_ nanoparticles in the bath gave the composite. The Zn composite coating showed better corrosion resistance characteristics than the pure Zn coating when observed through an anodic polarization curve. The electrochemical behavior of Zn–WO_3_ composites offers enhanced corrosion resistance compared to pure zinc coatings. This can be observed in Figure 10, Figure 11 and Figure 12.

**Figure 13:**
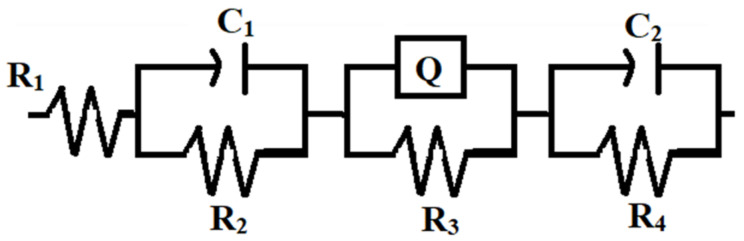
Equivalent circuit for the Zn and Zn–WO_3_ composite electrodeposit.

Detailed insight of the characteristics of the electrochemical process that occur at the electrode/solution interface in a corrosive media are driven through EIS measurements. Furthermore, it also explores the in situ and non-destructive probing relaxation mechanism with a large frequency range (FR). The EIS measurements on all coatings (Zn, and Zn–WO_3_ composite) at the open circuit potential (FR of 100 kHz to 0.1mHz) were carried out. The collected experimental data were represented in Nyquist plots, as shown in Figure 12. In Figure 12, curve (a) represents the Nyquist plot of the bare Zn deposit and curves (b) and (c) represent the Nyquist plots of Zn composites II and III, containing 0.5 and 1 g/L of WO_3_ particles. The Nyquist plots were recorded in 3.5% NaCl solution. Figure 13 shows the equivalent circuit corresponds to the fitted data of the Nyquist plots. 

Table 3 presents the various parameter values of the fitted curves, where R_1_, R_2_, R_3_ and R_4_ are the solution resistance, coating resistance, pore resistance and charge transfer resistance of the coatings, respectively. Terms C_1_ and C_2_ stand for the capacitance of the coating and capacitance of the double layer, respectively; Q is the constant phase element of the coating. From Figure 12, it can be observed that the semi-circle of the Zn composite containing 1.0 g/L has a larger diameter compared to the Zn composite obtained from 0.5 g/L and the bare Zn deposit. This shows that higher corrosion resistance for curve (c), i.e., composite deposit obtained from the 1 g/L of WO_3_ particles, compared to the other composite deposit and bare Zn deposit.

## 4. Conclusions

Currently, nickel and chromium composite coatings are applied to protect steel parts, but they are not generally recommended due to their hazardous nature. Furthermore, alternate to galvanizing practices attempt being made to apply environment-friendly coating material for protecting steel substrate useful for practical applications. The current study investigates the effect of adding WO_3_ particles into the Zn matrix by electrodeposition techniques. The following research findings are presented below:

1. The pure Zn and Zn–WO_3_ composites are electrodeposited successfully on mild steel specimens.
2. The Zn bath solution with 1 g/L of WO_3_ particles greatly influences the deposition process.
3. The WO_3_ particles were present and incorporated in the Zn matrix, which was observed from SEM analysis.
4. The incorporated WO_3_ particles changed the surface morphology of Zn deposits from a coarse grain to smaller grain size.
5. The microhardness of Zn and Zn–WO_3_ of 0.5 g/L and Zn–WO_3_ of 1 g/L composite electrodeposits are found equal to 47.6, 65.6 and 73.2 HV, respectively. An increase in WO_3_ particle concentration (from 0.5 g/L and 1.0 g/L) tends to increase the micro-hardness by 11.58% on electrodeposits. Electrodepositing WO_3_ particles of 1 g/L composites resulted in a 53.78% increase in microhardness compared to bare Zn coating deposits.
6. The corrosion rate of Zn and Zn–WO_3_ of 0.5 g/L and Zn–WO_3_ of 1 g/L composite electrodeposits is found equal to 32.69, 8.229, 2.803 Å min^−1^, respectively. The better corrosion resistance with composite coatings is attributed to the WO_3_ particles, which act as a physical barrier to the corrosion phenomenon.
7. Zn–WO_3_ composite coatings can be economically coated onto steel parts (tanks, containers, boilers, etc.) and for those applications that require higher corrosion resistance.

## Figures and Tables

**Table 1:** Optimized constituents of Zn bath solution and its operating parameters.

Deposit	Bath Solution	ZnSO_4_ (g/L)	Na_2_SO_4_ (g/L)	H_3_BO_3_ (g/L)	CTAB (g/L)	WO_3_ (g/L)	Operating Parameters
**I**	Zn	200	40	8	0.05	-	Current density: 4A dm^-2^
**II**	Zn composite	200	40	8	0.05	0.5	pH-3.0
**III**	Zn composite	200	40	8	0.05	1.0	Anode—Zn metal Cathode—mild Steel Stirring rate—300 rpm Cathode—40 mm × 40 mm × 1 mm Dimension Plating time—20 min

**Table 2:** Electrochemical coating parameters extrapolated from Tafel plots.

Samples	β_a_ (V^−1^)	β_c_ (V^−1^)	E_corr_ (V)	i_corr_ (A)	Corrosion Rate (Å min^−1^)
Zn	10.912	4.417	−1.068	1.148 × 10^−4^	32.69 ± 0.27
Zn–WO_3_ (0.5 g L^−1^)	17.583	5.771	−1.061	2.890 × 10^−5^	8.229 ± 0.22
Zn–WO_3_ (1.0 g L^−1^)	26.995	4.611	−1.033	9.844 × 10^−6^	2.803 ± 0.19

**Table 3:** Electrochemical parameters of the coatings derived from impedance spectra.

Samples	R_1_ (Ωcm^2^)	R_2_ (Ωcm^2^)	C_1_ (F)	R_3_ (Ωcm^2^)	Q	R_4_ (Ωcm^2^)	C_2_ (F)	Rp (Ωcm^2^)
Zn coating	9.014 × 10^−4^	76.50	5.626 × 10^−7^	537.6	3.586 × 10^−6^	139.5	4.323 × 10^−4^	753.6
Zn–WO_3_ (0.5 g L^−1^)	3.093 × 10^−11^	85.70	3.195 × 10^−7^	1733	14.86 × 10^−6^	920.7	3.123 × 10^−6^	2739.4
Zn–WO_3_ (1.0 g L^−1^)	4.769 × 10^−4^	624.2	0.714 × 10^−6^	3292	10.65 × 10^−6^	52.00	6.828 × 10^−7^	3968.2

## Data Availability

Data can be made available upon request.
